# Research on the supply-demand balance evaluation and driving mechanism of community public service facilities

**DOI:** 10.1371/journal.pone.0322109

**Published:** 2025-05-02

**Authors:** Jian Chen, Ying Fu, Shenglan Ma, Qiao Chen, Wanqing Zhang, Junlin Huang

**Affiliations:** 1 Hunan Sida Yuan Planning Consulting Research Co., Ltd, Changsha, China; 2 School of Geographical Sciences, Hunan Normal University, Changsha, China; Peking University, CHINA

## Abstract

The configuration level of community-level public service facilities is an important indicator for assessing the completeness of the urban public service system, and its spatial equilibrium directly impacts residents’ quality of life. This study takes 400 living circles in Changsha as samples and, based on the theory of “spatial equilibrium,” applies CRITIC method, system coordination model, and geographic detector to systematically evaluate the “supply-demand” equilibrium of community-level public service facilities. The study finds that: (1) The supply of public service facilities exhibits spatial agglomeration characteristics, while the demand distribution is significantly dispersed, with regional heterogeneity in the supply-demand matching; (2) There is a “Matthew effect” in the configuration of facilities, with significant equilibrium differences between the central urban areas and suburban regions; (3) The coverage of bus stops, farmers’ markets, and multifunctional sports fields are key driving factors, and transportation convenience and daily demand satisfaction are the core pathways to improving equilibrium. This study, by constructing a dynamic “supply-demand” adaptation framework, reveals the spatial equilibrium characteristics of community-level public service facilities and provides systematic planning ideas for optimizing urban public service resource allocation.

## Introduction

As a micro carrier of urban governance, the configuration effectiveness of community-level public service facilities profoundly impacts residents’ sense of access and well-being. Community-level public service facilities are not only the material carriers of residents’ daily lives but also the micro representation of social fairness and spatial justice. With the accelerated pace of urbanization and changes in population structure, residents’ demands for public services such as education, healthcare, and culture are increasingly diversified and differentiated. The traditional standardized configuration model based on indicators like “per thousand people” and “service radius” has become difficult to meet the dynamically changing needs. How to address the “supply-demand mismatch” problem and achieve precise facility layout responses has become a core issue in refined urban governance.

In China, the planning of community public service facilities is undergoing a paradigm shift from “supply-oriented” to “demand-oriented.” The early configuration model centered around the “per thousand people” indicator, which, while ensuring the quantity of facilities met certain standards, neglected the heterogeneity of population density, the diversity of behavioral needs, and the differences in spatial accessibility. This led to issues such as “facilities without services” and the coexistence of “inefficient supply and excessive agglomeration.” In recent years, with the promotion of the “living circle” concept, facility planning has embraced new ideas. Policies such as the “Community Living Circle Planning Technical Guidelines” attempt to bridge the time-space mismatch between facility supply and residents’ activities by defining “5-10-15 minute” service radius layers. However, current practices still face three major challenges: first, facility configuration largely relies on static population data, making it difficult to respond promptly to population mobility and changing demands; second, there is a lack of systematic tools for supply-demand matching evaluation, and quantitative analysis of spatial equilibrium is insufficient; third, the “Matthew effect” in facility configuration between suburban new districts and old urban areas exacerbates, limiting the overall service efficiency improvement.

In this context, this study uses 400 living circles in Changsha as the research sample, constructs a “supply-demand” coupling coordination model based on the theory of “spatial equilibrium,” aiming to break through the limitations of traditional planning. By integrating multidimensional methods such as CRITIC and geographic detectors, the study systematically analyzes the spatial agglomeration characteristics of facility supply, the trend of demand distribution’s decentralization, and their synergistic mechanisms. The focus is on addressing the following questions: (1) How can the spatial equilibrium of community-level public service facilities be quantitatively assessed? (2) What are the driving factors of supply-demand imbalance and their spatial differentiation patterns? (3) How should differentiated regions formulate facility optimization paths? The research results can provide methodological support for “people-centered” facility planning, help achieve equitable allocation and efficient utilization of public service resources, and provide methodological references for other cities globally. This will help cities more accurately assess the effectiveness of public service facility configurations and improve residents’ sense of well-being and belonging. This study emphasizes the importance of “spatial equilibrium” measurement and, through quantitative analysis, reveals the spatial characteristics of facility configurations and supply-demand matching, providing scientific basis for urban planning and policy-making.

## Literature review and research framework

### Literature review

With the advancement of China’s new urbanization strategy, the level of urban public services has significantly improved, and remarkable progress has been made in the equalization of basic public services. However, with the improvement of urban residents’ living standards and the rapid changes in the demand for public services, the problems of insufficient planning of public service facilities and the mismatch between supply and demand have become increasingly prominent, which have become the key factors restricting the high-quality development of cities [1]. Strengthening the effective supply and rational distribution of urban public resources has become the main task of urban construction [2]. Urban public service facilities are public facilities that provide public services such as education, medical care, and entertainment for urban residents [3] The allocation of public service facilities is a game between social justice and humanistic care [4]. As the basic unit of urban residents’ life and urban governance [5], the allocation level of public service facilities is directly related to the people’s sense of gain, happiness, and security. Under the concept of “people-oriented”, many cities have begun to pay attention to the community planning of 15-minute living circles in recent years [6,7]. To meet the diverse needs of residents, discussing the scientific and rational allocation of community public service facilities from the perspective of human demand has become an urgent scientific issue that needs to be addressed [8].

With the acceleration of urbanization and the improvement of residents’ quality of life, research on allocating community-level public service facilities has gradually become a focus of attention in academia and practice. Early research primarily adopted a sociological perspective, aiming to enhance the quality of life for residents by optimizing community development and social governance. These studies covered areas such as healthcare [9], infrastructure, environment [10], and sports [11], and were characterized by novel concepts [12,13], broad participation [14], and methodological innovation [15,16]. However, in the past two decades of rapid development in China, although the relevant research has increased, there are still obvious deficiencies in the depth of theory and breadth of application, and there are obvious problems with the characteristics of “technical tools” [17–19].

First, the existing literature largely focuses on macro-level analyses of the quantity and spatial distribution of facilities, with insufficient attention given to the individual needs of community residents and soft environment facilities (such as mental health support, cultural services, etc.) [20,21]. This hardware-oriented research approach neglects the impact of soft facilities on residents’ happiness and quality of life, leading to a lack of precision and humanized considerations in public service facility allocation schemes. Second, although some studies have attempted to introduce concepts of green, ecological, and humanized configuration, practical applications often emphasize the quantity and speed of facility development, failing to effectively balance sustainability and ecological benefits [22,23]. This strategy not only results in inefficient resource use but also contributes to environmental and social problems. Finally, while some studies have incorporated technical methods such as urban computing and econometric modeling into the scientific allocation process, most remain at the level of static data analysis, lacking the capacity to dynamically adjust to the rapidly changing needs of communities [24,25]. These shortcomings indicate significant theoretical and methodological gaps in current research on community-level public service facility allocation, particularly in the inclusion of soft environment facilities, the application of sustainable allocation concepts, and the development of dynamic allocation mechanisms.

To address the aforementioned challenges, this study introduces the concept of “spatial equilibrium” [26], aiming to develop a comprehensive supply-demand evaluation framework based on this theory, with the community life circle as the evaluation unit. This framework systematically assesses the equilibrium and adaptability of community-level public service facility allocation. “Spatial equilibrium” theory emphasizes the optimal spatial allocation of resources and the consideration of regional heterogeneity, enabling the rational distribution of resources and the coordination of economic and social activities [27,28]. The objectives of this study are threefold: first, to address the deficiencies in existing research related to soft facilities and demand-side analysis, specifically by incorporating community soft environments and residents’ personalized needs into the facility allocation evaluation framework, thereby achieving a people-centered and precise allocation. Second, the study integrates concepts of green, ecological, and humanized configurations, striving to develop facility allocation schemes that align with sustainable development goals and respond to residents’ needs for a high-quality living environment. Finally, by combining spatial equilibrium theory with dynamic urban computing and econometric modeling methods, the study constructs a dynamic evaluation framework capable of reflecting real-time changes in community needs, thus providing technical support for achieving dynamically balanced allocation of public service facilities. This scientific evaluation system not only provides an effective analytical approach for the scientific and balanced allocation of public service facilities at the community level in China, but also offers a methodological reference for other cities around the world. By focusing on the supply-demand system, it helps these cities more accurately assess the effectiveness of public service facility allocation, enhance the accessibility and fairness of public services, avoid resource waste and imbalances, and improve residents’ sense of happiness and belonging.

### Research framework

**Fig 1:**
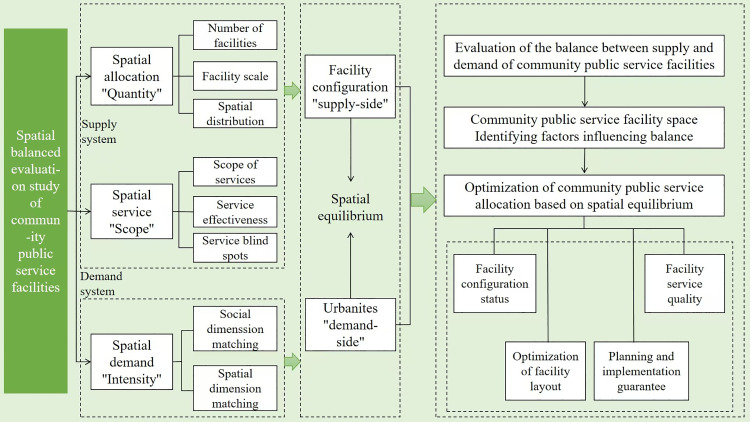
Research framework.

Spatial equilibrium theory emphasizes the dynamic balance between resource supply and demand in the spatial dimension, with the core concept being the optimization of spatial structure to maximize the overall efficiency of resource allocation. This study extends the theory to the field of community public service facilities and proposes a “Supply—Space—Demand” triadic synergy framework (Fig 1).On the supply side, the focus is on the quantity of facilities (“quantity”) and service coverage (“range”), quantifying the clustering and accessibility of facility distribution. Based on POI (Point of Interest) data and planning documents, 12 indicators are constructed from two dimensions: “Quantity” (facility density and size)And “Range”(Service radius and the proportion of service blind spots). On the demand side, the focus is on the residents’ behavior characteristics (“intensity”), combining human vitality and economic activity to represent dynamic demand. Using WeChat heat maps and consumption data, demand intensity is extracted based on: “Intensity”(Population vitality density and economic activity level). The Space Equilibrium Development Index (CD) is used to measure the degree of match between supply and demand in spatial units. The Geographical Detector model is applied to reveal key influencing mechanisms, supporting differentiated optimization strategies. By constructing a “Supply—Demand” coordination development model, this study integrates the CRITIC method and the Geographical Detector, overcoming the limitations of using a single indicator or static analysis, and providing methodological support for the precise optimization of community-level facility allocation.

### Indicator system

This study focuses on the “spatial equilibrium” dynamic adaptability theory analysis framework of “Supply-Space-Demand,” coordinating regional supply-demand balance and the coordination of key supply factors. It constructs an evaluation indicator system for the level of community public service facility allocation, forming quantifiable and comparable evaluation standards [29]. Based on the principles of scientificity, comparability, accessibility, and comprehensiveness, a spatial equilibrium development evaluation indicator system for community public service facilities is established (see Table 1). Among them, the “demand system” serves as one of the core dimensions of the evaluation system. Population status and economic development level are key driving factors for the demand system. Therefore, indicators such as population dynamics (e.g., population size, age structure, population inflow and outflow) and economic vitality (including per capital income, employment rate, industry development trends, etc.) are selected to represent the demand for community public service facilities. These indicators precisely depict the actual demand intensity and potential demand trends of different communities in terms of population and economic dimensions. The “supply system” comprehensively covers 9 core elements, including transportation, convenience services, and park green spaces, with a total of 28 specific indicators. These detailed and comprehensive indicators evaluate the supply level and spatial distribution characteristics of community public service facilities in all aspects, providing a solid data foundation and quantitative support for subsequent supply-demand balance evaluations. This ensures the scientificity and reliability of the evaluation results, enabling them to better serve the decision-making process in urban planning and community development.

**Table 1:** Evaluation index system for the allocation of community public service facilities.

Dimensional layers	Element layers	Specific indicator layers		Weights
Community public service facility provision system	Getting around	Transport facilities	Bus stop coverage	0.087
			Subway station coverage	0.033
			Branch road network density	0.081
			Density of parking facilities points	0.036
	Public service facilities	Educational facilities	Kindergarten coverage	0.056
			Primary school coverage	0.046
			High school coverage	0.041
		Medical facilities	Community health service station coverage	0.037
			Street health center coverage	0.030
			Hospital coverage	0.029
		Cultural facilities	Community cultural room coverage	0.003
			Coverage of cultural activity centers	0.016
			Coverage of cultural venues at the district level and above	0.038
		Sports facilities	Community multi-purpose sports field coverage	0.046
			Coverage of fitness activity centers for all	0.044
			Coverage of sports facilities at the district level and above	0.043
		Security facilities	Emergency shelter coverage	0.016
		Social welfare	Coverage of home care service stations	0.011
			Daycare center coverage	0.006
			Nursing home coverage	0.026
		Government services	Coverage of community public service centers	0.021
			Street office coverage	0.031
			Dispatch coverage	0.044
		Convenience services	Coverage of amenities	0.032
			Farmers’ market coverage	0.051
	Recreation and leisure	Park green	Street-level green space coverage	0.031
			Community park coverage	0.025
			Integrated park coverage	0.038
Community public service facility demand system	Demand dynamics	Population dynamics	Population vitality density	0.442
		Economic dynamism	Economic activity	0.558

*Note: The weight calculation method is described in section critic method.

## Methods

### Research methods and data

#### Study area.

**Fig 2:**
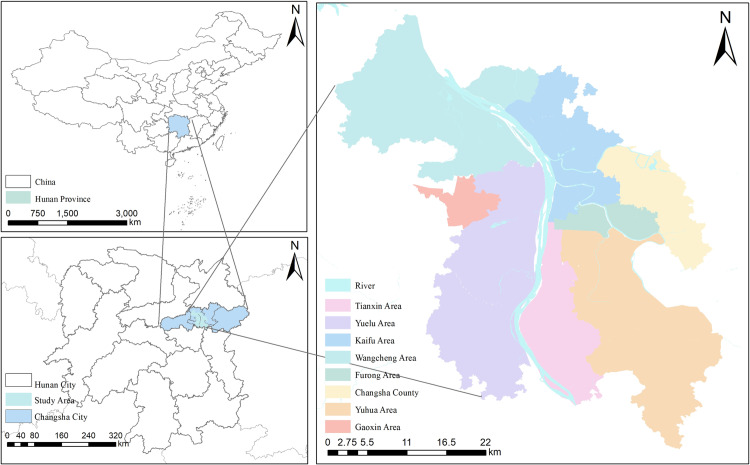
Study area.

In recent years, Changsha has actively responded to the national call for urban renewal, vigorously implementing a series of policies such as the renovation of old communities, upgrading of vegetable markets, and urban renewal. As a result of these efforts, the facility coverage rate in the central urban areas has been significantly improved, the city’s appearance has been refreshed, and the quality of life for residents has greatly improved. However, suburban new districts still face supply lag issues due to factors such as construction cycles, and there remains a gap between facility configuration and residents’ demands. This study takes 400 living circles in the central urban areas of Changsha as the basic research units. These living circles cover three typical regions: the central urban area, transition zones, and suburban new districts, as shown in Fig 2. By conducting an in-depth study of these three regions, this research can comprehensively and systematically reflect the spatial heterogeneity of community-level public service facility configuration in Changsha, revealing the differences between regions in terms of facility supply and demand, and providing strong support for subsequent analysis and planning.

#### Data sources.

This study integrates multi-source data to build a “supply-demand” evaluation database to comprehensively and accurately assess the supply-demand balance of community-level public service facilities in Changsha. POI data of community-level public service facilities in Changsha, including the names, types, and geographic locations of facilities, were obtained through the Gaode Map API (2023). Planning documents related to the boundaries of living circles, such as the city’s overall plan, land use plan, and community public service facility plans, were collected from the Changsha Natural Resources and Planning Bureau. WeChat travel heatmap data (2023) was used to reflect human activity patterns. Merchant ratings and consumption index data (2023) were obtained from Dianping, serving as indicators of economic vitality. Road network data were sourced from OpenStreetMap. All data underwent GIS spatial registration, topology checks, and normalization to ensure consistency in spatial analysis (Table 2).

**Table 2:** Data Sources and Processing.

Data Type	Data Source	Time Range	Spatial Processing
Living Circle Boundaries	Changsha Natural Resources and Planning Bureau	2023	Topology verification, projection transformation
Public Service Facility POI	Gaode Map API	2023	Kernel density analysis, service range modeling
Human Activity	Tencent Location Big Data (WeChat Heatmap)	2023	Raster aggregation to living circle units
Economic Vitality	Dianping	2023	Principal component analysis to construct a composite index
Road Network and Land Use	OpenStreetMap	2023	Network analysis, buffer zone generation

### Research methods

#### CRITIC method.

The study uses the range method to perform dimensionless processing on the raw data. The specific steps are as follows: First, the maximum and minimum values of each indicator’s data are identified, and the difference between them, i.e., the range, is calculated. Then, for each indicator’s data, subtract the minimum value from it, and divide the result by the range, which gives the standardized value of the data. After this processing, the data will fall within the [0,1] range, achieving dimensionless processing.

When the indicator is a positive one, the formula is:


$$x_{j}=\frac{x_{j}-x_{\min}}{x_{\max}-x_{\min}}$$ (1)

When the indicator is a negative one, the formula is:


$$x_{j}=\frac{x_{\max}-x_{j}}{x_{\max}-x_{\min}}$$ (2)

After standardizing the sample data using the above formulas (1) and (2), the standardized values of each indicator can be obtained. This study uses the CRITIC (Criteria Importance Through Intercriteria Correlation) method to calculate the weights of the indicator layer. The calculation process is as follows:

Let$\;S_{j}$ be the standard deviation of the j-th indicator, $\overset{―}{x_{j}}$ be the average value of the j-th indicator, and $V_{j}$ be the CRITIC weight of the j-th indicator.

(1) Calculate the mean value of the eigenvalue of each evaluation index:

$$\overset{―}{x_{j}}=\frac{1}{n}\sum_{i=1}^{n}x_{ij}(i=1,2,\cdot \cdot \cdot ,n;j=1,\;2,\;\cdot \cdot \cdot ,m)$$ (3)

In the formula,$X_{ij}$ represents the eigenvalue of the j-th evaluation indicator in the evaluation object; and n is the number of evaluation objects.

(2) Calculate the standard deviation of the characteristic value of the evaluation index:

$$S_{j}=\sqrt{\frac{1}{n-1}\sum_{i=1}^{n}{\left(x_{ij}-\overset{―}{x_{j}}\right)}^{2}}$$ (4)

(3) Correlation coefficient matrix between calculated indexes:

$$r_{jk}=\frac{\sum_{i=1}^{n}\left(x_{ij}-\overset{―}{x_{j}}\mathrm{\backslash rightleft}(x_{ij}-\overset{―}{x_{k}}\right)}{\sqrt{\sum_{i=1}^{n}{\left(x_{ij}-\overset{―}{x_{j}}\right)}^{2}\sum_{i=1}^{n}{\left(x_{ik}-\overset{―}{x_{k}}\right)}^{2}}}$$ (5)

$r_{jk}$ represents the correlation coefficient between indicator j and indicator k;$x_{ik}$ represents the eigenvalue of indicator k.

(4) Calculate the CRITIC weight of each evaluation index:

$$c_{j}=S_{j}\sum_{k=1}^{m}\left(1-r_{jk}\right)$$ (6)

$c_{j}$represents the CRITIC weight of evaluation indicator j; m is the total number of evaluation indicators.


$$\omega_{j}=\frac{{Vc}_{j}}{\sum_{j=1}^{m}c_{j}}$$ (7)

In the formula, $\omega_{j}$ represents the final weight of the evaluation indicator.

#### Coupling coordination degree model.

(1) The study uses the coupling coordination degree model (CCDM) to evaluate the spatial equilibrium of community public service facilities. The CCDM can reflect the degree of harmony and consistency between system elements and between systems, and the overall equilibrium level. The coupling degree model can be upgraded to the CCDM which is designed to indicate whether the elements have a healthy co-development relationship [30,31]. The formulas are as follows:


$$C={\left[\frac{U_{1}\times U_{2}}{{\left[\left(U_{1}+U_{2}\right)/2\right]}^{2}}\right]}^{\frac{1}{2}}$$ (8)

(2) Spatial Balance Development Index (CD). Given that the coordination degree only measures the coupling coordination between systems in a simplistic way, the study introduces a Development Index (D) to construct an index that can measure the overall balance and coordination utility of the supply and demand systems. Its calculation method is as follows:


$$D=\sqrt{C\times T\;}\;T=\alpha U_{1}+\beta U_{2}$$ (9)

where *U*_*1*_ and *U*_*2*_ are the comprehensive indices of supply and demand systems, respectively; *α* and *β* in the formula are generally taken as 0.5 [32,33]; This study believes that the supply and demand of public service facilities at the urban community level hold equal importance in residents’ lives, thus assigning them equal weights. This results in the calculation of the Spatial Balance Development Index (CD). By comparing the overall development levels between the two systems, it can be observed that the values are positively correlated with the development degree. Based on this, the spatial balance relationship between the supply system and demand system of community-level public service facilities can be accurately identified for each research unit, providing strong data support and theoretical basis for subsequent planning and decision-making.

#### Geographical detector.

The geographical detector (GD) is a statistical tool for identifying spatial variability in geographical objects and understanding their driving mechanisms [34]. This method is used to explore the driving factors that determine the spatial equilibrium of public service facility allocation. The equation is as follows:


$$q=1-\frac{\sum_{h=1}^{L}N_{h}\sigma_{h}^{2}}{N\sigma^{2}}=1-\frac{SSW}{SST}\;(h=1,2,3\ldots L)$$ (10)

where *q* falls between [0–1], the bigger the value, the stronger the explanatory ability of the driver on the spatial equilibrium of public service facility allocation. The stratification of each indicator factor is denoted by *h*. *N*_*h*_ and *N* are the sample sizes for stratum h and the region as a whole, respectively; $\sigma_{h}^{2}\;$and$\;\sigma^{2}$are the variances for stratum h and the overall region, respectively.

## Results

### Analysis of supply and demand allocation levels and spatial characteristics

**Fig 3:**
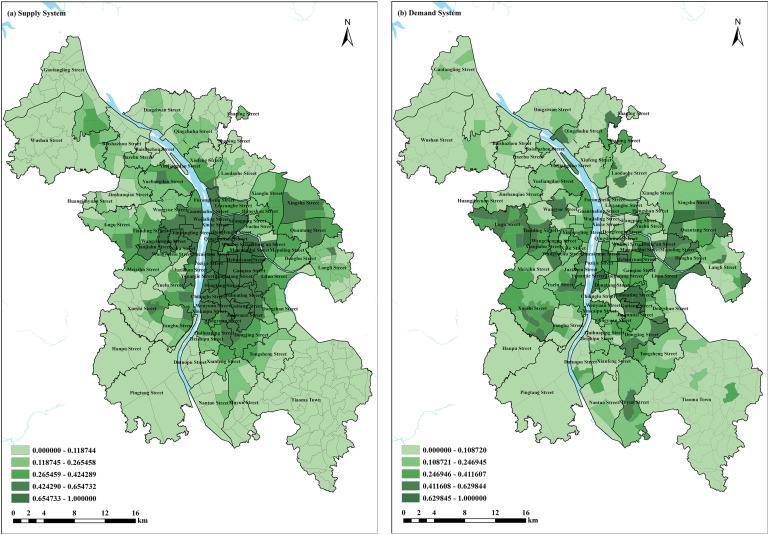
Changsha community-level public service facility supply system and demand system scoring results.

Through an in-depth statistical analysis of the supply and demand systems of community-level public service facilities in Changsha, the spatial differentiation characteristics are revealed, mainly manifested in the following three aspects:

(1) The spatial differentiation of supply is driven by the dual mechanisms of “stock renewal” and “new city expansion,” as shown in Fig 3(a). The old city area, centered around Wuyi Square, forms a high-density supply core, with education and medical facility coverage rates reaching 92% and 85%, respectively, confirming the effectiveness of urban renewal policies in Changsha. This area exhibits a “vertical composite” feature in facility allocation, with each building on average supporting 3.2 types of services. However, there is a problem of functional redundancy, such as a 68% overlap in the 500m service range of community health service stations. A new supply corridor has emerged along the subway line, where the facility density within an 800m buffer zone around stations (0.78/km²) is 2.3 times that of peripheral areas. However, there is a “heavy emphasis on transportation, light on livelihoods” tendency — the coverage of bus stations (76%) is significantly higher than that of farmers’ markets (43%) and elderly care facilities (29%), which is disconnected from the population influx demand under Changsha’s “rail urban” strategy. The accessibility of facilities outside the city’s ring road is only 35% of that in the central urban area, with medical facility blind spots accounting for 41%, forming a significant mismatch with the “eastward expansion and westward linkage” spatial strategy, exposing lagging support issues in new city construction.
(2) The demand distribution shows a “function-oriented discrete” characteristic as shown in Fig 3(b). Urban centers (such as the Wuyi business district) and emerging functional nodes (such as Meixi Lake International New City) form demand hotspots, with both population density and economic activity being high. This reflects the nighttime service demand driven by the “moonlight economy” (for example, the nighttime usage intensity of the Fisherman’s Wharf restaurants is three times that of the daytime). The transitional area along both banks of the Xiangjiang River shows a gradient of declining demand intensity, with the demand density of the old city area on the east bank significantly higher than that of the new city area on the west bank, highlighting the insufficient functional integration between the two banks under the “cross-river development” strategy. The demand index in the suburban areas is generally below 0.3, with public services depending on cross-regional commuting (average commuting time increased by 22 minutes), exacerbating the “pendulum” supply-demand contradiction.

Changsha’s “strong center + axial expansion” spatial strategy and residents’ “multi-centered” behavioral demand create structural conflicts. First, there is an imbalance between the two banks of the Xiangjiang River: the supply on the east bank exceeds that on the west, but the demand on the west is growing much faster than on the east, expanding the mismatch due to differing growth rates. Second, the metro effect is differentiated: within the 1km radius of subway stations, there is a coexistence of “supply surplus—demand overflow,” such as the cultural facilities around the Orange Island station having a usage rate of only 32%, while the commuting population relies 71% on the farmers’ markets 3km away. Lastly, there is a delay in policy response: the “15-minute living circle” coverage rate is 89%, but the facility allocation standards in suburban new districts still follow the central urban model, resulting in a 55% service blind spot for small community parks (<1hm²), which is disconnected from the “micro-space” demand of residents.

In summary, the supply and demand system of community-level public service facilities in Changsha sketches a spatial differentiation map with strong local characteristics and clear problem orientation. These characteristics not only precisely identify the pain points and challenges currently faced by facility allocation but also provide solid empirical support for the precise implementation of future planning and management strategies. They guide the city’s resource allocation towards a more balanced, efficient, and human-centered direction, laying a solid foundation for the sustainable development of Changsha and similar cities.

### The spatial effects of supply and demand equilibrium

**Fig 4:**
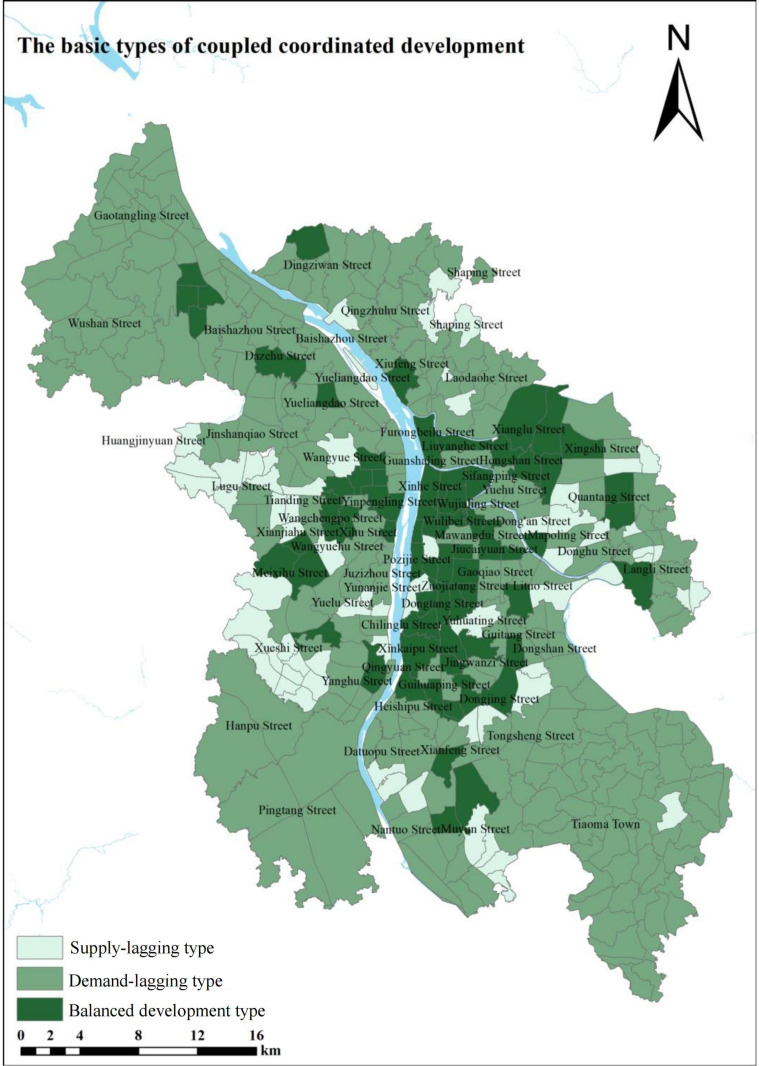
Results of calculating the basic types of coupled and coordinated development of public service facilities in the community in Changsha. .

Based on the calculation and analysis of the Spatial Balance Development Index, the supply and demand equilibrium of community-level public service facilities in Changsha shows significant spatial differences.(As shown in Fig 4). The analysis results indicate that the spatial distribution of community-level public service facilities in the central urban area of Changsha exhibits notable spatial differentiation characteristics. The balanced development life circles are mainly distributed at the urban periphery, totaling 81, accounting for 20.25%; demand-lagging life circles are mainly concentrated in the city center, totaling 180, accounting for 45%; and supply-lagging life circles total 139, accounting for 34.75%. From this, it can be seen that there is a significant spatial mismatch between the supply and demand of community-level public service facilities in the central urban area. The city center shows a significant demand-lagging effect, which calls for supply-side reforms to improve the efficiency of community-level public service facility allocation. To scientifically represent the spatial equilibrium characteristics of public facilities within life circles, this study uses the “seven-part method” to construct a classification system for the spatial balance development of the supply and demand systems, and establishes corresponding evaluation criteria [35–37], as shown in Table 3.

**Table 3:** Spatial equilibrium degree classification system and evaluation criteria.

Spatial equilibrium degree	Subcategory	d(y)with s(x) the relationship between	Basic type
0.800~1.000	Quality equilibrium development category	d(y)>s(x)	Supply system lagging type
		d(y)=s(x) ^*^	Synchronous type
		d(y)<s(x)	Demand system lagging type
0.700~0.800	Good equilibrium development category	d(y)>s(x)	Supply system lagging type
		d(y)=s(x) ^*^	Synchronous type
		d(y)<s(x)	Demand system lagging type
0.600~0.700	Intermediate equilibrium development class	d(y)>s(x)	Supply system lagging type
		d(y)=s(x) ^*^	Synchronous type
		d(y)<s(x)	Demand system lagging type
0.500~0.600	Primary equilibrium development category	d(y)>s(x)	Supply system lagging type
		d(y)=s(x) ^*^	Synchronous type
		d(y)<s(x)	Demand system lagging type
0.400~0.500	Mild disequilibrium development category	d(y)>s(x)	Supply system lagging type
		d(y)=s(x) ^*^	Synchronous type
		d(y)<s(x)	Demand system lagging type
0.300~0.400	Moderate disequilibrium development category	d(y)>s(x)	Supply system lagging type
		d(y)=s(x) ^*^	Synchronous type
		d(y)<s(x)	Demand system lagging type
0.000~0.300	Severe disequilibrium development category	d(y)>s(x)	Supply system lagging type
		d(y)=s(x) ^*^	Synchronous type
		d(y)<s(x)	Demand system lagging type

Note: If the difference is less than 2%, the object is considered to be of the type where the demand system is synchronized with the supply system.

**Fig 5:**
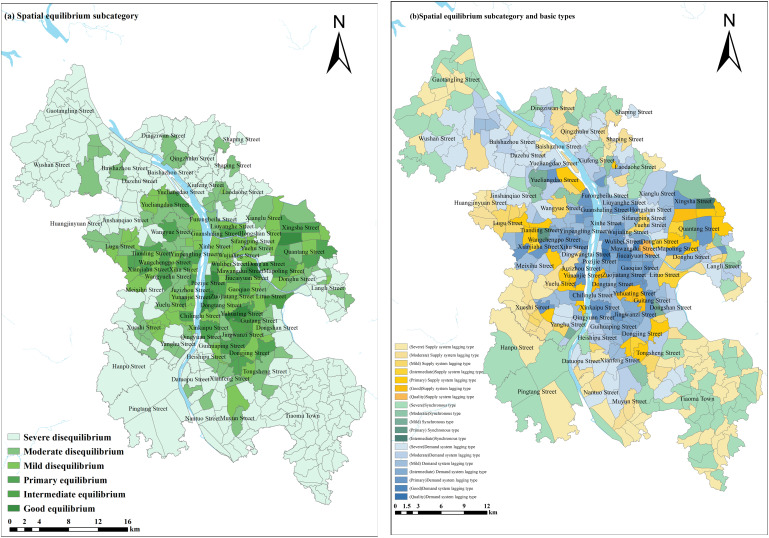
Calculation results of the coupling coordination development index of community-level public service facilities in Changsha.

The results show that the spatial misalignment characteristics of community-level public service facilities in Changsha are significant, as shown in Fig 5(a). The coordination degree of the supply and demand system for community-level public service facilities in Changsha (with an average C index of 0.856) is significantly higher than the overall development level (with an average D index of 0.176), indicating that while the overall development level still needs improvement, certain achievements have been made in supply-demand coordination. Although the community-level public service facilities in the urban periphery have a relative advantage in terms of coordination in allocation, the spatial distribution of the CD index still shows significant centralization. Specifically, areas with high CD index values are mainly concentrated in the core functional areas of the city, such as Pozi Street, while low-value areas are concentrated in transitional zones of urban functional clusters, such as Datuopu Street. The overall facility allocation effect on the east bank of the Xiangjiang River is better than that on the west bank, with regions such as the southern part of Liuyang River, the northern part of the ring expressway, and the western part of Huangxing Avenue showing good facility spatial balance and forming a multi-cluster central structure centered around several streets. The facility allocation level along the Yuelu Avenue on the west bank of the Xiangjiang River is higher, but it decreases towards the north and south sides along the transportation axis.

It can be seen from Fig 5(b), Changsha’s community-level public service facilities overall present a “T-shaped” spatial pattern with a “core-periphery” characteristic, consistent with the overall spatial development structure of the city. This study’s findings are similar to related research conclusions, confirming the universality of supply-demand spatial misalignment. However, under the rapid urbanization context in Changsha, influenced by the dual-track mode of “new city expansion — old city renewal,” the “Matthew Effect” is more pronounced. That is, the central urban area continues to attract population agglomeration due to the facility stock advantage, while the new areas, constrained by construction cycles, have supply lagging behind demand growth. This suggests that Changsha not only has spatial mismatches in the supply and demand of community-level public service facilities but that these mismatches are more pronounced in the central urban area compared to new districts, further exacerbating spatial imbalance.

In summary, community-level public service facilities in the central urban area of Changsha are relatively well distributed, but optimizing population distribution is necessary to avoid resource waste. Peripheral areas need to increase facility construction and promote facility sharing to improve facility allocation levels and meet the actual needs of residents.

### Spatial balance driving mechanism

The CRITIC method is used to detect the impact of internal factors on the overall system’s coordinated development. The indicator with the highest weight selected by the CRITIC method includes the following factors: the bus station coverage rate in the transportation facilities category, the elementary school coverage rate in the education facilities category, the community health service station coverage rate in the medical facilities category, the coverage rate of cultural venues above the district level in the cultural facilities category, the community multi-functional sports field coverage rate in the sports facilities category, the police station coverage rate in the government service category, the farmers’ market coverage rate in the convenience services category, the comprehensive park coverage rate in the park and green space category, and the population density in the demand vitality category. A total of 9 indicators are selected as the detection factors in the geographical detector.

The influence of each detection factor on the spatial equilibrium of public service facilities at the community level calculated by the GD model is shown in Table 4, and the importance of each factor affecting the spatial equilibrium development of public service facility allocation varies significantly. The factors of bus stop coverage, farmers’ market coverage, and community multi-purpose sports field coverage dominate, while other factors are relatively less important. The effect of bus stop coverage on the spatial equilibrium of public service facility allocation is significantly higher than other factors, with a q-value of 0.679. Public service facilities supply and demand coupling to achieve primary equilibrium and above the circle of life, public transport station coverage of more than 50% of all. In 2018, the Ministry of Transport announced that 12 cities, including Changsha, were awarded as “National Public Transport City Construction Demonstration Cities”, and in 2018, Changsha’s public transport motorized travel sharing rate reached 54.2%. A reasonable layout of bus stops can greatly meet the activity needs and facility demands of residents, thus promoting an equilibrium allocation of public service facilities. The coverage of farmers’ markets significantly influences the spatial equilibrium of public service facility allocation, with a q-value of 0.613 and an explanatory power exceeding 60%. Among various public service facilities related to livelihoods, residents depend most directly on farmers’ markets. In Shanghai’s 15-minute life circle plan, it is recommended that elderly daily facilities be centered around vegetable markets, encouraging the creation of public activity spaces for community residents. The coverage of community multifunctional sports fields has an explanatory power of 57.5%, indicating a substantial impact on spatial equilibrium. Life circles with sports field coverage of 50% or more achieve balanced development in public service facility allocation. Population vitality density, a key indicator of the demand system, also plays a role, with a q-value of 0.356. Human demand is the fundamental driving force behind the development of coupled supply and demand for public service facilities. As the subject of the coupled system of supply and demand of public service facilities, the change of people’s demand causes the population to agglomerate and spread, thus having an impact on the spatial equilibrium development of public service facility allocation.

**Table 4:** Impact of elements to spatial equilibrium degree (q) of community public service facilities.

	Bus stop coverage	Primary school coverage	Community health service station coverage	Coverage of sports facilities at district level and above	Community multi-purpose sports field coverage	Dispatch coverage	Farmers’ market coverage	Integrated park coverage	Population vitality density
q statistic	0.679	0.458	0.474	0.483	0.575	0.531	0.613	0.346	0.356
p-value	0.000	0.000	0.000	0.000	0.000	0.000	0.000	0.000	0.000
Policy Implications	Promote the construction of rail transit centers	Promote the sharing of facilities and enhance community public service network	Refine community public service network	Advance the hierarchical sharing between the city, district, and community.	Promote the development of multi-functional sports facilities.	Enhance through the sharing of community facilities.	Improve the community’s commercial service network.	Establish a network of blue and green spaces for service.	Enhance a comprehensive, talent-friendly service network.

**Fig 6:**
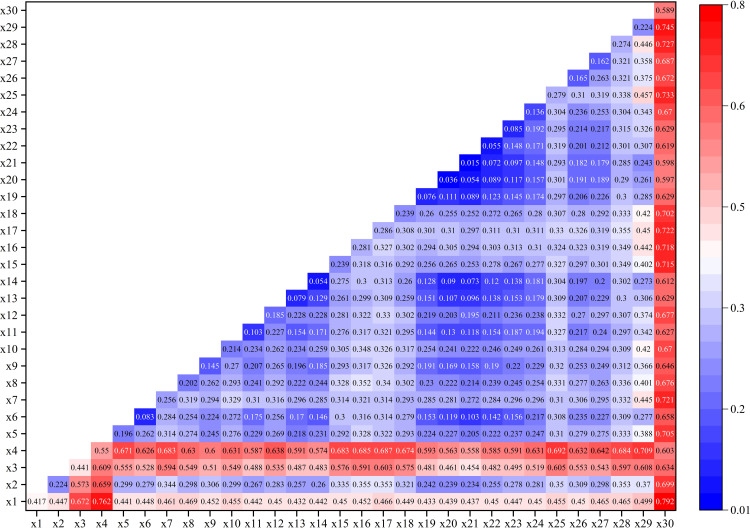
Mutual factor detection results.

From Fig 6, it can be observed that the 30 influencing factors of the spatial equilibrium of community-level public service facilities in Changsha do not independently affect regional economic disparities, but instead exhibit interactive effects. The explanatory power significantly increases when any two factors interact, and the interaction types are classified as either dual-factor enhancement or nonlinear enhancement. There are no instances of weakening or independent relationships, indicating that the formation of spatial imbalance in community-level public service facilities in Changsha is the result of the combined effects of multiple factors.

In terms of the interaction capacity of the influencing factors, economic vitality (X30), public transportation station coverage (X1), road network density (X2), and parking facility density (X4) have the most prominent interaction capabilities among the 30 factors, with explanatory power above 60%. Among these, economic vitality (X30) demonstrates a synergistic enhancement effect when interacting with any other factor, showing the greatest explanatory power. The next strongest interaction capabilities are found with parking facility density (X4), road network density (X3), and public transportation station coverage (X1), all of which have explanatory power above 50%. This suggests that the spatial equilibrium of public service facilities in Changsha is mainly shaped by the interaction of traffic infrastructure, economic vitality, and other factors. The primary reason for this is that the improvement of economic vitality not only drives investment growth, promotes employment and consumption, but also directly fosters the construction and upgrading of public service facilities, laying a solid foundation for spatial equilibrium. A high coverage of public transportation stations greatly enhances the accessibility of facilities, reduces residents’ travel costs, and promotes balanced utilization of facilities. An increased road network density improves the road network,

*In the Fig 6, X1 to X30 represent the 30 indicators constructed in the indicator system, which are as follows: Bus stop coverage, Subway station coverage, Branch road network density, Density of parking facilities points, Kindergarten coverage, Primary school coverage, High school coverage, Community health service station coverage, Street health center coverage, Hospital coverage, Community cultural room coverage, Coverage of cultural activity centers, Coverage of cultural venues at the district level and above, Community multi-purpose sports field coverage, Coverage of fitness activity centers for all, Coverage of sports facilities at the district level and above, Emergency shelter coverage, Coverage of home care service stations, Daycare center coverage, Nursing home coverage, Coverage of community public service centers, Street office coverage, Dispatch coverage, Coverage of amenities, Farmers’ market coverage, Street-level green space coverage, Community park coverage, Integrated park coverage, Population vitality density, Economic activity.

## Discussion

This study explores in depth the underlying contradictions faced by the allocation of community-level public service facilities under the “material form planning” mindset, specifically the disconnect between facility supply and dynamic demand, a problem that further exacerbates spatial imbalances. Through the integration and innovation of existing technologies and theories, this study proposes a comprehensive and practical evaluation framework for the allocation of community public service facilities. Compared with the previous studies, by introducing the theory of “spatial equilibrium”, this study expands the focus from a single hardware facility configuration to a comprehensive evaluation considering soft environment facilities and individual needs, emphasizing the interdependence, influence, and coordinated development degree between demand and supply, which not only improves the scientific and operational nature of the assessment but also makes it easier to connect the assessment results with the actual administrative jurisdiction boundary, thus providing an operational reference for planning practice. Through empirical analysis of Changsha City, the following main findings and insights were obtained:

(1) Theoretical Breakthrough: From “Static Supply” to “Supply-Demand Coupling”. Unlike traditional planning, which focuses on the static logic of “per thousand people,” this study proposes a “supply-demand” dynamic adaptation framework based on the “spatial equilibrium” theory. The results show that facility supply exhibits a “core-area concentration” characteristic, while demand, due to population mobility and economic vitality differences, presents a “multi-center dispersed” distribution pattern (Fig 5). This discovery validates the ubiquity of the supply-demand spatial mismatch, similar to studies conducted in numerous cities, yet the “Matthew effect” in Changsha is particularly pronounced， likely linked to its dual-track model of “new city expansion—old city renewal” under the rapid urbanization context. The study shows that relying solely on facility quantity standards cannot achieve efficient resource allocation; a system coordination model is needed to quantify the “people-land-facility” interaction, providing tool support for refined governance.
(2) Practical Insights: Differentiated Pathways to Address the “Matthew Effect”. The imbalance between the central urban area and suburban new districts reveals the “siphoning effect” in resource allocation: mature areas continue to attract population concentration due to the advantage of facility stock, while new districts, constrained by construction cycles, face supply lagging behind demand growth. In response, planning should adopt differentiated strategies: First, cross-river collaboration should be strengthened, with complementary functions on both sides of the Xiang River, where the eastern bank focuses on optimizing facility sharing, and the western bank enhances the response speed of basic service provision. Second, rail empowerment should be implemented, promoting “TOD + public service” integrated development around metro stations, with priority given to filling gaps in farmers’ markets and elderly care facilities. Third, flexible adaptation should be established by creating a dynamic “demand-supply” matching index for new city construction, incorporating population migration forecasts into facility allocation standard revisions.
(3) Driving Mechanism: Priority of Facilities Based on Daily Demand. The results from the geographic detector show that bus stops (q=0.679), farmers’ markets (q=0.613), and multifunctional sports fields (q=0.575) are the core driving factors for spatial equilibrium. This finding aligns with the current perspective proposed by some cities that the “15-minute living circle should focus on basic services,” but further quantifies the weight of transportation convenience and daily needs. Based on the factor analysis results, differentiated policy measures are formulated to ensure the coverage of the above-mentioned facilities, as shown in Table 4. These measures prioritize ensuring the coverage of these facilities, while also using big data for real-time monitoring of demand changes to avoid “planning failure.”
(4) Policy Alignment: From “Balanced Allocation” to “Happiness Perception” Although Changsha has been repeatedly recognized as one of the “most happiness-oriented cities,” this study reveals that there are still significant spatial disparities in the balance of facilities (Fig 4). Future policies should break away from the conventional mindset of “balance equals equality” and incorporate “soft indicators” such as residents’ happiness and usage efficiency into the evaluation system. This will guide the shift in facility allocation from “quantity standardization” to “experience optimization.”

In general, this study, based on the theory of spatial equilibrium, provides a highly valuable and systematic approach to analyzing urban resource allocation issues. The research clearly emphasizes that future urban planning should proactively address the systemic contradictions between individual needs and public space resource allocation. By implementing a dynamic adjustment mechanism for the “supply-demand” balance, the layout of public service facilities can be optimized and upgraded to better meet the increasingly diverse needs of urban residents. Ultimately, this will maximize overall benefits and provide strong support for the prosperity of the city and the well-being of its residents.

## Conclusions

This paper takes Changsha as the research subject and closely follows the “spatial equilibrium” theory to explore the spatial utility measurement methods for community-level public service facilities. The layout planning of facilities in other cities provides valuable and beneficial experiences for reference. The paper draws the following main conclusions:

(1) By constructing a “supply-demand” system coordination model, this study reveals that the spatial equilibrium of community-level public service facilities in Changsha exhibits characteristics of “central lag and peripheral inefficiency.” The central urban area needs to improve shared usage rates through functional integration, while the suburban new districts should strengthen transportation and daily facility provision. This method effectively avoids the drawbacks of traditional planning practices, which often start with neighborhood centers, ensuring that the evaluation of living circle units better aligns with actual administrative boundaries. It provides more precise and detailed data support for planning departments and lays a solid theoretical foundation for similar research in the future, with significant academic value and practical implications.
(2) By integrating geographic detectors and CRITIC method, this study identifies the coverage of bus stops, farmers’ markets, and sports fields as key driving factors, providing a quantitative basis for the timing and prioritization of facility construction. The research deeply reveals the spatial imbalance issues in the current facility layout and its underlying mechanisms, emphasizing the need to address differentiated demand and supply across regions during the planning process. It provides crucial support for subsequent planning adjustments.
(3) A “monitoring-assessment-feedback” dynamic mechanism is established. The central urban area focuses on stock renewal and smart transformation, while suburban new districts adopt “demand forecasting + flexible planning” and simultaneously improve cross-regional facility sharing policies.

This study, based on Changsha as a sample, offers a replicable analytical framework for facility planning in similarly structured cities. Future research could further explore multi-scale (such as block-community linkage) and multi-actor (such as government-market collaboration) equilibrium pathways, combining digital twin technology for dynamic simulation of resource allocation. Against the backdrop of accelerated urbanization, the goal is to address the imbalance in urban resource allocation through scientific, rational, precise, and effective planning methods. This not only provides scientific basis for Changsha’s future facility planning and optimization but also offers feasible solutions for other cities to address uneven distribution of public service resources and mismatched population distribution with facility layouts. This will promote further development of global urban public service facility planning, contributing to fairer and more sustainable urban development, which has become a key task in enhancing the overall competitiveness of cities and residents’ well-being.
