# Correction: Research on the supply-demand balance evaluation and driving mechanism of community public service facilities

**DOI:** 10.1371/journal.pone.0338719

**Published:** 2025-12-09

**Authors:** Jian Chen, Ying Fu, Shenglan Ma, Qiao Chen, Wanqing Zhang, Junlin Huang

In Fig 2, the dashed line at the southernmost part of China’s territorial waters was inadvertently excluded from the map of China. Please see the correct Fig 2 here.

**Fig 2:**
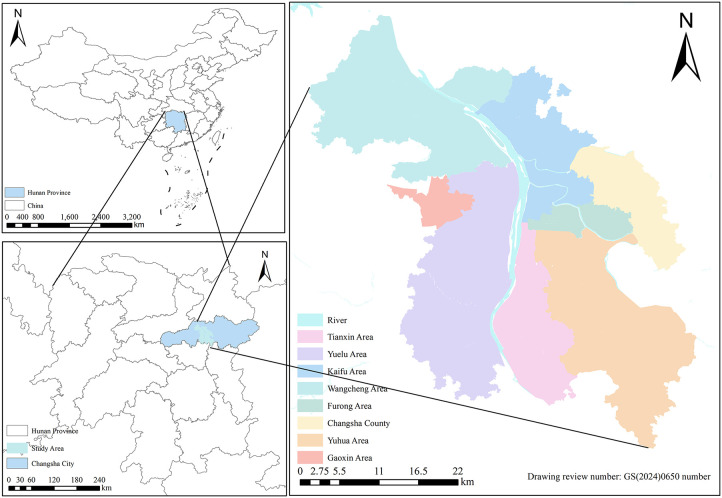
Study area.

## References

[1] Chen J, Fu Y, Ma S, Chen Q, Zhang W, Huang J. Research on the supply-demand balance evaluation and driving mechanism of community public service facilities. PLoS One. 2025;20(5):e0322109. doi: 10.1371/journal.pone.0322109 40315219 PMC12047832

